# Sex Bias in Autoimmunity: New Findings and New Opportunities

**DOI:** 10.1016/j.xjidi.2025.100391

**Published:** 2025-06-20

**Authors:** Vincent van Drongelen, Joanna Rew, Allison C. Billi

**Affiliations:** 1 Department of Dermatology, Michigan Medicine, University of Michigan, Ann Arbor, Michigan, USA

**Keywords:** Autoimmunity, Sex

## Abstract

Autoimmune diseases result from the immune system’s inability to discriminate between self and foreign antigens, leading to development of self-reactive immune cells and autoantibodies that can cause organ damage and failure. It has long been known that many autoimmune diseases are more common in females. Although much progress has been made over the years, the exact mechanisms for this sex bias are not yet fully understood. In this review, we provide an overview and update on how chromosomes, genes, sex hormones (including gender-affirming hormone therapy), immunometabolism, and the skin can play a role in sex-biased autoimmunity. We also identify gaps in our understanding that require additional research. In an era of increased development of personalized medicine, a thorough understanding of sex bias in autoimmunity may facilitate the development of much-needed targeted therapeutics, thereby reducing the risks of broader immunosuppression and adverse effects that lead to premature termination of treatment by patients.

## Introduction

For the immune system to function adequately, it must be able to distinguish between self and nonself. Through a process called major histocompatibility complex (MHC) self-restriction, the immune system is trained to recognize self-antigens that are expressed on the surface of all cells and are encoded by the MHC locus, also known as the HLA locus in humans. Inability to make this distinction between self and nonself results in misdirection against the organism’s own cells, leading to damage of that which it is supposed to protect. This is the fundamental mechanism underlying autoimmune and autoinflammatory diseases. Although various genes and genetic variants have been identified that play a role in development of autoimmune diseases, these diseases are generally believed to be the result of a complex interaction between genetic predisposition (genetics) and environmental and/or biological factors. Globally, 4% of people are estimated to have at least 1 autoimmune disease (Sohn, 2021). In the United States, that percentage is estimated to be about double, with over 23.5 million people affected (National Academies of Sciences, Engineering, and Medicine, 2020). The continuing increase in prevalence of autoimmune diseases is a significant public health concern owing to the subsequent burden on healthcare systems, families, and communities.

Following early observations made by Philip S. Hench in the 1930s (Hench, 1938), when rheumatoid arthritis (RA) disease improved during pregnancy, links between biological sex and the distribution or severity of systemic chronic inflammatory or autoimmune diseases have repeatedly been reported. It is now well-established that of all the patients who suffer from autoimmune disease, 75–80% are women (Collison, 2018). Common autoimmune diseases that show a particularly high female predominance include RA and systemic lupus erythematosus (SLE), with female-to-male ratios of 11:1 and 9:1, respectively (Cooper and Stroehla, 2003). Interestingly, these female-to-male ratios vary according to age of disease onset; for example, in childhood-onset SLE, female-to-male ratio is 3:1–5:1, whereas in adult-onset SLE, the ratios increase to 10:1–15:1 (Cattalini et al, 2019).

Although this increased incidence in females has been known for many decades, females have historically been excluded from biomedical studies and clinical trials owing to the potential effects of hormonal fluctuations on data (Klein and Morgan, 2020). This has resulted in a discrepancy in our understanding of the role of sex in autoimmune disease development. Recently, more attention has been given to sex bias in autoimmune diseases in clinical trials and health care. Strategies are being developed on how to adequately leverage this variable to reduce adverse effects and improve treatment response. Currently, sex specificity is a factor seldom considered in treatment, and there is little sex-specific health care. This is, for example, relevant for psoriatic arthritis, an autoimmune disease that has similar prevalence in men and women. However, women with psoriatic arthritis have higher disease activity, higher levels of pain, and lower functional capacity score than male patients and thereby a need for sex-specific adjustment of treatment approach (Passia et al, 2022).

The precise explanation of why females more frequently develop autoimmune diseases than males is currently a subject of extensive investigation. Increasing our understanding about the role of sex in autoimmune disease development could have significant potential for future therapeutic interventions. In this paper, we discuss several factors that could contribute to these sex differences in autoimmune disease development as well as recent developments and areas that require future exploration.

Before we discuss the role of sex bias in autoimmune disease development, it is important to define what is meant by ‘sex’ and ‘gender.’ Sex refers to biological factors including reproductive organs (testes or ovaries), levels of sex hormones (testosterone and estrogen), and the presence of sex chromosomes (X and Y chromosomes). By contrast, gender refers to sex-related behavior or lifestyle factors and is not binary. It is important to note that sex and gender are not mutually exclusive and can intersect to affect disease outcomes. An example of gender influence on disease is the exposure of workers in male-dominated professions, such as stone masonry or painting and decorating, to environmental toxins, such as silica or vinyl chloride, which have been implicated in the pathogenesis of systemic scleroderma (SSc) (Abbot et al, 2018; Black et al, 1983; Fox and Kang, 1992; Freire et al, 2024). Both sex and gender can influence the outcome of a disease, which make it difficult to distinguish and identify how each contributes to disease development.

## Differences in the Immune System Between Females and Males

**Figure 1:**
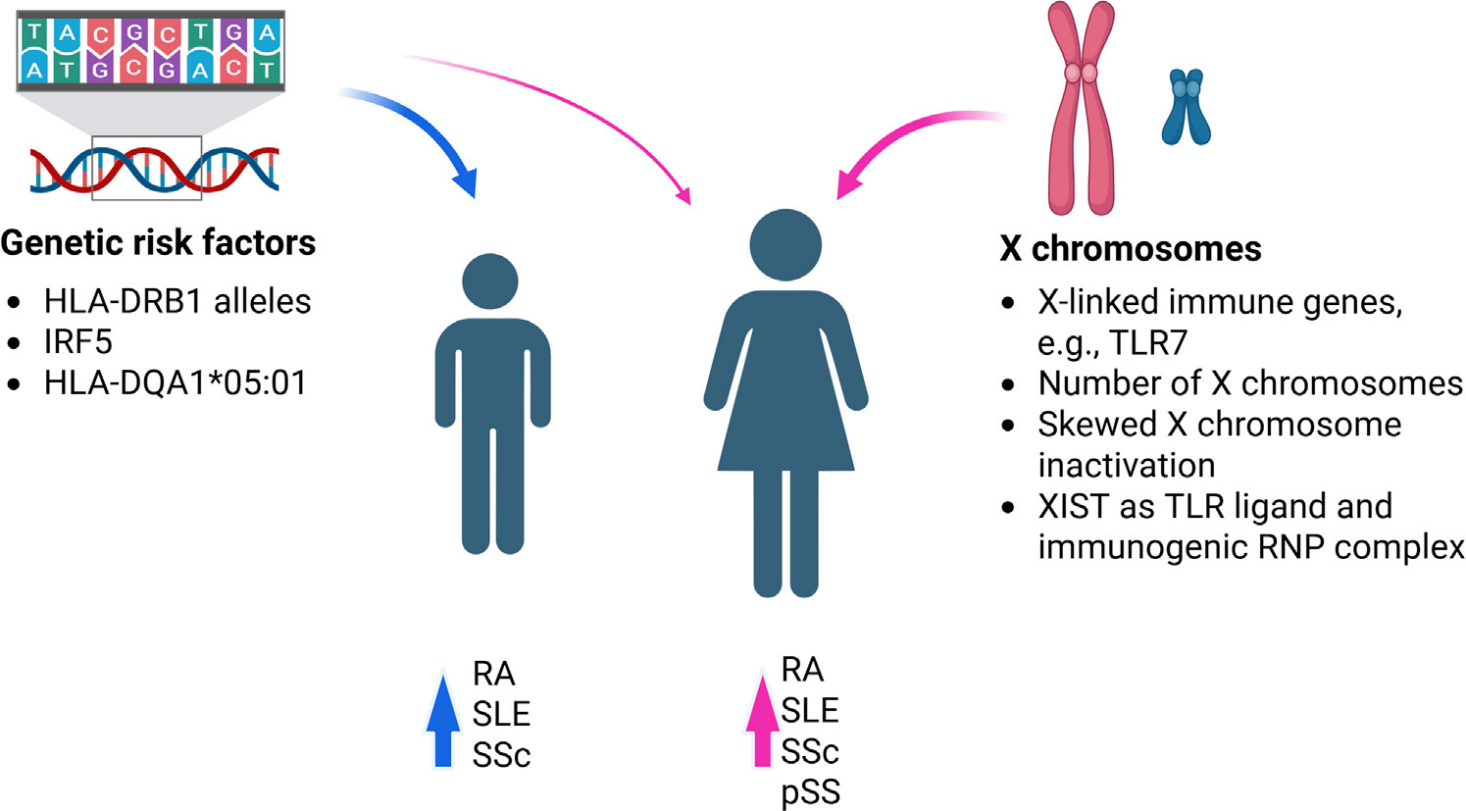
**Influence of genetic risk factors on sex bias in autoimmune disease.** Examples are indicated by the bulleted items. The text provides more details. Image was created with bioRender. pSS, primary Sjogren syndrome; RA, rheumatoid arthritis; RNP, ribonucleoprotein; SLE, systemic lupus erythematosus, SSc, systemic sclerosis; TLR, toll-like receptor; XIST, X-inactive specific transcript.

It is well-established that females and males have inherent differences in their immune systems with regard to cellular composition and function. This can partly be attributed to genetics (Figure 1) and partly to endocrine factors. Males have a predisposition to be more susceptible to infections and cancer development owing to an overall weaker immune response, whereas females generally have stronger innate and adaptive immune responses, which has a significant survival advantage when fighting off infections. The drawback of this “advantage” is the approximately fourfold higher risk of autoimmunity (Kronzer et al, 2021).

Lymphocytes (B cells and T cells) are a group of white blood cells that have a central role in protecting the body by regulating the immune response through the production of antibodies and elimination of cells presenting antigens identified as nonself. These nonself antigens may be pathogen derived or altered, as frequently occurs in tumor cells. Because of their central role in the immune system, lymphocytes also play a role in the development and progression of autoimmune diseases. Defective immune tolerance mechanisms, such as defective MHC self-restriction, lead to the immune systems’ inability to distinguish self from nonself, resulting in the expansion of autoreactive T cells and autoantibody-producing B cells. Organ damage occurs as a consequence of both direct recognition of self-antigens and indirect mechanisms, such as formation of antigen–antibody immune complexes that deposit in tissues and trigger an inflammatory cascade. In addition, as discussed further below, lymphocytes express receptors for sex hormones both at the cell surface and in the cytosol that mediate different transcriptional and epigenetic changes to alter immune signaling and function. Cell surface receptor sex hormone signaling can activate cytoplasmic signaling and rapid calcium fluxes, which lead to rapid activation of signaling pathways and widespread changes in T-cell gene expression and function (Bereshchenko et al, 2018; Brown and Su, 2019). Nuclear receptor sex hormone signaling in immune cells leads to upregulation of numerous key immune-related genes, including those encoding IL-1β, TNF, IFNy, and chemokines (Klein and Flanagan, 2016).

## Genetics, Genes, Sex Chromosomes, and X Chromosome Inactivation in Autoimmune Diseases

### Genetic variants that increase the risk for development of autoimmune diseases

Much investigation into the basis of female sex bias in autoimmunity has focused on performing GWASs to identify genetic variants in the form of single-nucleotide variations (SNVs) associated with increased disease risk. Associations between autoimmune diseases and the HLA locus on chromosome 6 were among the earliest discovered, and they remain the strongest autoimmune disease risk associations identified to date (Matzaraki et al, 2017). This highly polymorphic locus consists of 3 regions: class I, class II, and class III. Chromosome 6 encodes 6 HLA genes—*HLA-A*, *-B*, and *-C* (class I) and *HLA-DR*, *-DP*, and *-DQ* (class II)—and 132 genes encoding proteins that have important roles in the immune system such as TNF; complement factors C3, C4, and C5; and heat shock proteins (class III) (Shiina et al, 2009). Class I molecules present endogenous peptides to CD8+ T cells, and class II molecules present exogenous peptides to CD4+ T cells. Given the many genes encoded by this small segment of the human genome, it is not surprising that it has been associated with >100 different diseases, including autoimmune diseases such as RA, SLE, SSc, and psoriasis (Shiina et al, 2009). Interestingly, this association varies according to sex and is generally stronger in men than in women, even in diseases that demonstrate strong female sex bias such as RA and SLE. One example is the strong association between RA and *HLA-DRB1* alleles that contain the so-called “shared epitope” (Gregersen et al, 1987; van Drongelen and Holoshitz, 2017), a 5-amino-acid sequence in the HLA-DRB1 molecule that is associated with RA. Individuals with this high-risk genotype (*HLA-DRB1∗04:01* and *HLA-DRB1∗04:04*) have a 26-fold higher risk for RA than those without shared epitope alleles (MacGregor et al, 1995). Remarkably, the risk increases to 90-fold in men and doubles when disease onset is at age <30 years (MacGregor et al, 1995). Similarly, men who develop SLE have a higher cumulative genetic risk than women, especially regarding the HLA region and the *IRF5* gene (Hughes et al, 2012). Another example of male sex bias and HLA genes occurs in SSc. In a study with a small number of individuals, *HLA-DQA1∗05:01* was found to be more frequently carried by men with SSc than by parous women with SSc (Lambert et al, 2000). These examples demonstrate that men have a higher risk of carrying HLA susceptibility alleles for autoimmune diseases. However, the overall female predominance in autoimmune diseases suggests a role for other predisposing factors.

### Contributions of X chromosome–encoded genes and X chromosome dosage

Although GWASs have extensively been used to identify genetic loci associated with autoimmune disease (Khramtsova et al, 2019), the X chromosome has commonly been excluded from final analyses because sex-specific analyses require additional considerations as well as special tools and algorithms (Keur et al, 2022; König et al, 2014). Albeit less prominent, in juvenile autoimmune diseases such as juvenile SLE, juvenile Sjögren’s syndrome (SS), and juvenile dermatomyositis, female sex bias is also observed (Cattalini et al, 2019). Because these juvenile autoimmune diseases develop when males/boys and females/girls have similar levels of serum sex hormones, this suggests a role for sex chromosomes themselves in development of sex-biased autoimmune diseases. The X chromosome contains approximately 1100 genes. Most of the protein-coding genes on the X chromosome are unrelated to sex, and approximately 10% of the genes on this chromosome are involved in immune functions. In contrast, the Y chromosome has around 100 genes, of which most are related to sex determination and spermatogenesis, with the remainder being X chromosome homologs located in the so-called pseudoautosomal regions (PARs) such as *DDX3X*/*DDX3Y*, *USP9X*/*USP9Y*, and *KDM5C*/*KDM5D* (Cabrera Zapata et al, 2022; Goodfellow et al, 1983; Olney et al, 2020).

Immune genes on the X chromosome include toll-like receptor *(TLR)7*, *TLR8*, *FOXP3*, and *CD40L*. TLRs are a family of 10 evolutionarily conserved proteins that are important for recognition and defense against microbes. In the skin, activation of TLR7 has been implicated in several inflammatory skin diseases, including psoriasis (Kim et al, 2024), rosacea (Huang et al, 2023), and SLE (Yokogawa et al, 2014). The role for TLR7 in SLE pathogenesis has been well-established, and currently, TLR7 is being evaluated as a drug target for SLE treatment (Fukui et al, 2018; Souyris et al, 2019; Wang et al, 2022). One potential explanation for a mechanism could be that activation of TLR7 leads to the production of IFNα by plasmacytoid dendritic cells (pDCs), and this can be enhanced by 17β-estradiol in females. However, a recent study found that nonhematopoietic cellular sources rather than pDCs are responsible for IFN production prior to clinical autoimmunity (Psarras et al, 2020). Sequence variants affecting *TLR7* could also contribute to increased IFNα, effects that are possibly mediated by IRF5. Although this has been shown in mice, it has not been fully clarified in humans. Variants in *IRF5* itself have also been linked to SLE pathogenesis. Interestingly, men with SLE have a higher *IRF5* risk allele frequency (Hughes et al, 2012), and in patients with SLE, a strong association between *IRF5* risk variants and higher serum IFNα levels was found (Niewold et al, 2008). The link between TLR7 and SLE was recently further strengthened by identification of a child with severe SLE carrying a novel *TLR7* gain-of-function variant that, when introduced into mice, caused murine SLE (Brown et al, 2022). The variant was found to increase affinity of TLR7 for endogenous ligands, thereby promoting TLR7 activation and survival of B cells that recognize self-antigens.

In addition to genetic variants located on the X chromosome, the number of X chromosomes has been associated with higher risk or susceptibility to developing autoimmune diseases, such as SLE, SS, or SSc (Miquel et al, 2023). The influence of the X chromosome on development of autoimmune disease is demonstrated in men with Klinefelter syndrome. These individuals carry an additional X chromosome (47, XXY) and have a 14-fold increased risk for developing SLE and a 38-fold higher risk for developing primary SS (pSS) than men without Klinefelter (46, XY) (Harris et al, 2016; Scofield et al, 2008). Furthermore, women that have triple X syndrome (47, XXX) have a 2–3-fold higher risk for pSS and SLE than women with an XX karyotype (Liu et al, 2016).

Sophisticated mouse models have been developed in an effort to dissect the contributions of sex chromosomes, sex hormones, and the interaction of the 2 to sex-biased and sexually dimorphic diseases. The Four Core Genotypes model uncouples the mouse sex chromosome complement from gonadal sex by moving the testis-determining gene *Sry* to an autosome, enabling generation of mice that are XX with ovaries, XX with testes, XY with ovaries, and XY with testes (De Vries et al, 2002). This model has been used to examine how sex chromosomes contribute to sex bias in various phenotypes and diseases, including autoimmune diseases such as SLE (Arnold, 2020; Sasidhar et al, 2012). However, the Four Core Genotypes model was recently found to include an X–Y translocation that alters dosage of *Tlr7* and several other X chromosome genes, thus complicating interpretation of results involving this model (Panten et al, 2024). Another system termed the XY∗ model entails an aberrant PAR on the Y chromosome that recombines with the X chromosome to yield mice approximating XX and XO with ovaries and XY and XXY with testes (Eicher et al, 1991). This system has likewise been used in investigating the influence of sex chromosomes versus sex hormones in murine autoimmune disease (Voskuhl et al, 2018).

In summary, the existence of sex bias in juvenile autoimmune diseases implicates factors other than sex hormones and suggests a role for sex chromosomes in sex-biased autoimmunity. Approximately 10% of the genes encoded by the X chromosome are involved in immune functions, including *TLR7*, and increased number of X chromosomes has been associated with higher risk of autoimmune disease. Mouse models that uncouple chromosomal and gonadal sex may provide further insight into the roles of sex chromosomes and hormones in autoimmunity.

### X chromosome inactivation and a role for XIST

Under homeostatic conditions, one of the X chromosomes is silenced in females in a random manner in a process called X chromosome inactivation (XCI). Under normal circumstances, this results in 50:50 maternal versus paternal XCI. However, in autoimmune diseases such as SSc, this XCI is skewed, and over 80% of the cells show inactivation of the same X chromosome. XCI is heterogenous across different cell types, tissues, and individuals, and about 25% of X- linked genes are suspected to escape XCI, including *IRAK1*, *MECP2*, *CD40L*, *TLR7*, and *TLR8*. Interestingly, the inactive X chromosome seems to be predisposed to partial reactivation in lymphocytes in females, resulting in increased expression of X-linked immune genes that augment the risk for development of autoimmune disease such as SLE (Wang et al, 2016). The exact mechanism for the reactivation remains to be investigated.

This skewed XCI has stimulated considerable investigation into XCI as a potential cause of autoimmune diseases. New evidence suggests that *XIST* (X-inactive specific transcript), a long noncoding RNA (lncRNA) essential to XCI, may contribute to female predominance of SLE and, possibly, other autoimmune diseases (Andrade, 2024). Of note, *XIST* is expressed by the inactivated X chromosome and therefore not in XY males. One study by Crawford et al (2023) found *XIST* RNA to be a rich source of TLR7 ligands that activate pDCs to produce IFNα. They also established a likely unidirectional causal role of *XIST* inducing IFNα production by showing that *XIST* levels were not increased when SLE cells were treated with IFNα (Crawford et al, 2023).

An alternative hypothesis for the role of *XIST* in sex-biased autoimmunity is that *XIST*-containing ribonucleoprotein (RNP) complexes are immunogenic and promote autoimmunity. Male mice engineered to overexpress transgenic *XIST* are prone to autoimmunity, developing severe multiorgan pathology after transgenic *XIST* induction as well as autoantibodies against XIST–RNP complexes (Dou et al, 2024). This study also identified various novel autoantibodies against the XIST–RNP complex in sera from patients with SLE, SSc, and dermatomyositis. These studies have identified a novel sex hormone–independent mechanism for sex bias in autoimmunity, and future studies will be needed to assess whether these *XIST* RNP autoantibodies can aid in stratification of the disease states or patients.

To summarize, altered XCI may contribute to sex bias in autoimmune disease by skewing toward inactivation of a particular X chromosome and a predisposition to partial X chromosome reactivation in lymphocytes in females. Recent discoveries have identified important potential roles for the lncRNA *XIST* in autoimmunity, further implicating XCI as a driver of this sex bias.

## The Effects of Sex Hormones on Sex Bias in Autoimmune Diseases

Historically, the field was divided among those who believed that the X chromosome was the culprit driving sex bias in autoimmune disease and those who attributed the bias to sex hormones. Given the finding that the X chromosome contains many immune response genes and the fact that estrogen receptors (ERs) regulate some of these genes, there is likely an interplay between the 2. For example, studies have shown that in SLE, genetic variants of *ESR1* (which encodes ERα) are linked to SLE susceptibility (Xie et al, 2019). Another relevant example is that estrogens increase the expression of TLR7 and TLR8, important regulators of type I IFNs and known players in SLE (Young et al, 2014). This illustrates the complexity of studying the precise mechanisms through which genes and sex hormones contribute to autoimmune disease development, and this topic remains an area of intensive research.

### How sex hormones contribute to sex-biased autoimmune disease development

Sex hormones—broadly, estrogens, androgens (testosterone), and progesterone—are steroid hormones that are lipid soluble and can interact with receptors on the surface of target cells or with cytosolic receptors that primarily act by translocating to the nucleus to influence gene expression, although they can also exert effects through nongenomic mechanisms (Matsumoto et al, 2013). Furthermore, sex hormones can affect gene expression on the epigenetic level by influencing DNA methylation and chromatin conformation (Fullwood et al, 2009; Nugent et al, 2015). Gene expression analysis from various nonreproductive tissues indicates the existence of a sex-specific influence on transcription and that chromatin organization is a major determinant of sex-biased chromatin accessibility and gene expression (Brooks et al, 2014; Melé et al, 2015; Sugathan and Waxman, 2013).

Hormonal effects can also be concentration dependent. Estrogens can either be proinflammatory (at low concentrations) or anti-inflammatory (at high concentrations) and, thereby, have an important role in regulating the immune response. On the other hand, androgens and progesterone mainly have suppressive effects on the immune response. Moreover, in both males and females, there is a shared steroid conversion pathway between androgens and estrogens that is activated in inflamed tissue. In this pathway, a steroid aromatase converts androgens into estrogens. This could help explain the propagation of local inflammatory diseases in men (Castagnetta et al, 2003). To complicate things further, the presence of inflammatory cytokines such as TNF, IL-1, and IL-6 can stimulate the activity of aromatases, enzymes required for estrogen biosynthesis. This shows that inflammation itself can affect local estrogen levels and thereby control the inflammatory response (Herrmann et al, 2002).

### Effects of sex hormones on various immune cell types

The effects of sex hormones can vary depending on the hormone and which cell type is targeted (Table 1). Sex bias in the immune system arises from differential gene expression in the relevant immune cell subsets of males and females, and sex hormones influence this differential expression. Transcriptional profiling of human innate and adaptive immune cells—including monocytes, naive B cells, and various T-cell subsets—identified about 1875 transcripts that exhibit sex-biased expression, the majority of which were autosomal (Schmiedel et al, 2018).

**Table 1:** Effects of Sex Hormones on Immune Cell Function and Skin

Immune Cells	Estrogens	Androgens	Progesterone
Macrophages	Promotes anti-inflammatory response through cytokine production, chemotaxis, and phagocytosis; inhibits NF-kB pathway; increases TLR7 and TLR8 expression; reduces TLR4 expression; inhibits proinflammatory cytokine production; enhances M2 macrophage polarization and wound healing	Enhances macrophage migration and anti-inflammatory cytokine production	Inhibits anti-inflammatory response; inhibits TLR4 and TLR9 activation; impairs NF-kB activation
Dendritic cells	Promotes cell differentiation; promotes proinflammatory cytokine production; reduces IL-23 production; enhances T-cell stimulation	Decreases T-cell stimulation; decreases secretion of proinflammatory cytokines	Decreases the secretion of proinflammatory cytokines
Neutrophils	Enhances neutrophil activation; enhances cytokine and chemokine production; enhances cell survival; enhances NO synthase response		
NK cells	Enhances NK cell number; inhibits NK infiltration, cytotoxicity, and proliferative capacity		Enhances NK cell numbers
T cells	Enhances Th17 responses; enhances Th1 responses	Inhibits Th17 responses; inhibits Th2 responses and memory; inhibits Th1 responses; enhances Treg responses; inhibits lymphopoiesis; increases the expression of AIRE for elimination of self-reactive T cells	Inhibits CD4+ and CD8+ proliferation; enhances Treg responses; inhibits Th1 responses; enhances Th2 responses; inhibits Th17 responses
B cells	Enhances proliferation, differentiation, activation, and apoptosis; enhances somatic hypermutation and class-switch recombination; increases antibody production	AR signaling KO increases frequency of B cells; AR signaling KO increases antibody production	Increases the frequency of marginal zone B cells during pregnancy; decreases proportions of plasmablasts; increases the frequencies of plasma cells
Skin	ERβ is most prominent ER in the skin; is anti-inflammatory through repression of NF-κB; enhances expression of proinflammatory mediators; promotes keratinocyte proliferation, collagen deposition, and wound healing	Anti-inflammatory effects in cells with high AR expression, including keratinocytes; stimulates keratinocyte proliferation during wound healing and epidermal barrier formation; enhances keratinocyte migration in vivo	Anti-inflammatory through repression of NF-κB; decreases collagenolysis; promotes keratinocyte proliferation

Abbreviations: AIRE, autoimmune regulator; AR, androgen; ER, estrogen receptor; KO, knockout; NO, nitric oxide; Th, T helper; TLR, toll-like receptor; Treg, regulatory T cell.

Table was adapted from Hoffmann et al (2023). Details and additional references are provided in the main text.

Various immune cells, such as T cells, B cells, dendritic cells (DCs), monocytes, macrophages, and NK cells, as well as cells at skin and mucosal barrier sites express sex hormone receptors. The best-characterized sex hormone receptors are the intracellular receptors, which include ERα and ERβ, progesterone receptor (PR), and androgen receptor (AR) (Brown and Su, 2019). Activation of ERα leads to an anti-inflammatory response. For example, in macrophages, ERα activation promotes M2 macrophage polarization that facilitates wound healing (Campbell et al, 2014).

Variation in the levels of sex hormones can also lead to sex-biased differences in the expression of immune genes and subsequent immune response. For example, ERs act as transcription factors that mediate long-range chromatin interactions and form complexes at gene regulatory elements, thereby promoting epigenetic changes and transcription (Kovats, 2015). Although the effects of activation of ER on expression of type II IFNs have been described (Khan and Ansar Ahmed, 2015; Nakaya et al, 2006), the various potential mechanisms through which estrogen may affect type I IFNs is still being debated (Dong et al, 2015; Kovats, 2015; Singh et al, 2021). The concentration-dependent and context-dependent effects of estrogen are also apparent in various autoimmune diseases. In RA and multiple sclerosis, high estrogen levels during pregnancy have been implicated in decreased disease activity (Aggelakopoulou et al, 2016; de Man et al, 2008; Garnier et al, 2018). However, the effects of estrogen abundance are not so straightforward because, for example, in SLE, disease activity often increases during pregnancy (Baer et al, 2011).

Androgens have anti-inflammatory effects on immune responses in vitro and in vivo through various mechanisms. In the innate immune system, they reduce the expression of genes regulated by the proinflammatory transcription factor NF-κB (Cutolo and Straub, 2020). Androgens also reduce the secretion of proinflammatory cytokines such as TNF, IL-1β, and IL-6 from monocytes and macrophages and reduce lymphocyte proliferation, decreasing antibody and cytokine production (Fish, 2008; Gubbels Bupp and Jorgensen, 2018; Traish et al, 2018; Trigunaite et al, 2015). Furthermore, androgens directly affect the production of AR-positive B-cell precursors but not AR-negative mature B cells (Trigunaite et al, 2015). Androgens also have an important role in central tolerance by regulating the thymic expression of autoimmune regulator (AIRE), a transcription factor that is important for the elimination of self-reactive T cells. Androgens recruit ARs to the AIRE promoter and enhance its transcription in male mice and humans, whereas estrogens inhibit AIRE expression (Dragin et al, 2016; Zhu et al, 2016). In addition, androgens can also affect the expression of FOXP3, a transcription factor with a central role in the development of CD4+ regulatory T cells (Tregs). Evidence exists that during the ovulation phase of the menstrual cycle, androgens can increase FOXP3 expression in Tregs but that androgens have no effect on Tregs from males (Walecki et al, 2015). Currently, it is still unclear whether female predominance in many autoimmune diseases is a result of higher levels of female hormones such as estrogen, low levels of male hormones such as testosterone, or a combination of the 2.

To summarize, sex hormones directly influence immune cells by binding membrane-bound and/or intracellular receptors through which they mediate genomic and nongenomic effects. Although estrogens can have pro or anti-inflammatory effects, androgens and progesterone are generally anti-inflammatory. The concentration-dependent and context-dependent effects of estrogen have been shown to play a role in various autoimmune diseases, but the effects of systemic estrogen and other hormone levels on autoimmune disease risk and activity are nuanced and still under investigation.

### Investigating autoimmunity risk among transgender and gender-diverse individuals

Given the effects sex hormones have on the immune system and their potential to influence the course of autoimmune disease development, it is of interest to assess the effects of hormone therapy on the immune system and how this affects development or remission of autoimmune diseases. This is especially relevant for the transgender and gender-diverse (TGD) community, for whom gender identities and/or gender expressions do not necessarily align with the sex assigned at birth. It is estimated that this population comprises 0.3–4.5% of the population globally (Coleman et al, 2022). These individuals may receive gender-affirming hormonal therapy (GAHT) to acquire body changes that are concordant with their gender identities. GAHT can consist of feminizing/demasculinizing (typically oral or transdermal estradiol and androgen-lowering medication) or masculinizing (testosterone) therapy (Coleman et al, 2022; Hembree et al, 2017).

A recent prospective study followed a cohort of 54 GAHT-naïve TGD adults (29 assigned female at birth [AFAB], 25 assigned male at birth [AMAB]) for 3 years after initiation of GAHT. This modestly sized cohort did not demonstrate significantly increased risk of development of autoantibodies or autoimmune disease among AMAB or AFAB individuals during the first 3 years of GAHT (Marconi et al, 2025). However, a previous study examining a larger cohort of 146 transgender people—including individuals both receiving and not receiving GAHT—showed that 36% of AFAB trans men and 31% of AMAB trans women were positive for antinuclear antibodies (ANAs), significantly higher than in the general population (Ramos et al, 2020). Authors proposed multiple possible contributors to this elevated ANA positivity beyond prior or current GAHT exposure that might differentially affect the transgender community, including environmental exposures, trauma, infections, silicone implants, and other cosmetic procedures (Ramos et al, 2020). Nonetheless, the discrepancies in these early studies highlight the importance of carrying out large-scale, longitudinal prospective studies of TGD people to assess the long-term effects of GAHT on the presence of autoantibodies and development of autoimmune diseases.

Although these studies followed transgender people for development of autoantibodies, the effects of GAHT on the immune response have not been intensively investigated yet. A recent publication that followed 23 transgender men aged 18–37 years during the first 12 months of testosterone treatment found multiple immunological effects of gender-affirming testosterone treatment (Lakshmikanth et al, 2024). Although total white blood cell counts were unaffected by testosterone treatment, attenuation of type I IFN expression and reduced *IRF7* expression by pDCs were observed. Testosterone treatment was also associated with increased TNF responses by lipopolysaccharide-treated monocytes as well as increased expression of SLAMF7, a cell surface receptor that recently was described to potentiate TNF responses in macrophages (Simmons et al, 2022). NK cells were more prone to IFNy production after testosterone treatment. Several immunological sex differences were unaffected by testosterone treatment: although CD4/CD8 T-cell ratios were reported higher in female than in male participants (Lee et al, 1996), these ratios did not decrease in AFAB individuals who received GAHT in the form of testosterone therapy, nor did shifts in CD4+ T-cell polarization occur. This suggests that some aspects of immunological sex bias are regulated at the chromosomal level rather than by sex hormones. Although this report is among the first to study the effects of GAHT on the immune system so extensively and in great detail, several questions remain. For example, are the observed differences due to higher testosterone levels or indirect effects of lower estrogen levels? In addition, whether and how these changes contribute to protection against autoimmune diseases is currently unknown. As personalized medicine continues to develop in the future, understanding how hormone treatments contribute to or lower the risk for development of autoimmune diseases will be important, especially for those with a genetic predisposition.

Thus, the effects of GAHT on development of autoimmune disease remain incompletely resolved, with conflicting reports in the literature. A recent study with AFAB individuals treated with testosterone therapy identified some shifts in immunological sex differences with GAHT and some that remained unaffected. Large-scale, longitudinal prospective studies are needed to determine whether GAHT affects the development of autoimmune diseases.

## The Role of Sex Hormones in Skin and Inflammatory Skin Disease

In addition to their direct effect on the immune system, there is an abundant amount of information on the effects of nuclear sex hormone receptor signaling in the skin (Clocchiatti et al, 2016). This is of particular relevance for autoimmune diseases with prominent or primary skin involvement, including SSc, SLE, and isolated cutaneous lupus erythematosus (CLE).

### ER signaling in the skin

Both ERs, ERα and ERβ, are expressed in various skin cells. In general, ERβ is considered the primary ER expressed in the skin, with highest expression noted in epidermal keratinocytes, dermal fibroblasts, and various cell types within blood vessels as well as outer root sheath, epithelial matrix, and dermal papilla cells within the hair follicle (Thornton et al, 2003; Verdier-Sévrain et al, 2006). In contrast, ERα is mainly expressed by the sebaceous glands (Pelletier and Ren, 2004; Thornton et al, 2003). Estrogens regulate several aspects related to skin function, including proliferation, collagen deposition, wound healing, and inflammation (Verdier-Sévrain et al, 2006). As discussed previously, estrogens mediate both proinflammatory and anti-inflammatory functions, and the net effect depends on both the (immune) cell type mediating the response and which ER is involved. Similar to their effects on immune cells, ERβ activation results in the expression of proinflammatory mediators (Bereshchenko et al, 2018; Wilkinson and Hardman, 2017; Wu et al, 2022). Conversely, the anti-inflammatory effects of estrogens are primarily mediated through suppression of NF-κB signaling (Bereshchenko et al, 2018; Wilkinson and Hardman, 2017).

### AR and PR signaling in the skin

ARs are expressed in the cytoplasm of different skin cell types, including keratinocytes, fibroblasts, endothelial cells, and cells of the dermal papilla. The binding of androgens to ARs induces several effects on the different skin cell populations, including the regulation of inflammatory responses (Pace and Werz, 2020). In particular, androgens exert a primarily anti-inflammatory effect by inducing a direct reduction of the inflammatory process in cells that express high levels of ARs, including keratinocytes (Pace and Werz, 2020; Traish et al, 2018). The specific role of androgens on keratinocyte function is still controversial and under investigation. Although one study showed that testosterone stimulates keratinocyte proliferation during wound healing and epidermal barrier formation (Traish et al, 2018), in vitro experiments indicated a negative effect of androgens on keratinocyte migration. On the contrary, in vivo studies highlight an enhancement in the migration process. This suggests that such positive effects on migration require interaction between keratinocytes and other skin cell populations, such as fibroblasts (Lai et al, 2012).

As with ERs, 2 PRs exist: PR-A and the larger PR-B. Whereas PR-A is nuclear localized, PR-B can show both nuclear and cytoplasmic localization (Asavasupreechar et al, 2020). Progesterone primarily exerts anti-inflammatory and immunosuppressive activities in the skin through the repression of NF-κB, thereby suppressing the production of proinflammatory cytokines. Furthermore, as in the case for estrogens, progesterone regulates dermal collagen by decreasing matrix metalloproteinase activity in fibroblasts (Kanda and Watanabe, 2005) and promotes keratinocyte proliferation (Im et al, 2000).

Given the importance of keratinocytes in skin immunity and the impact of sex hormones in regulating keratinocyte proliferation and differentiation processes, it is worth exploring how hormonal imbalance can contribute to various immune-mediated inflammatory or autoimmune skin diseases. It is currently not well-understood how sex hormones’ effects on skin contribute to autoimmune disease development. A recent literature review found that there remains a debate regarding the use of hormonal contraceptives and hormone replacement therapy in individuals with autoimmune skin conditions (Lopes Almeida Gomes et al, 2024). However, given the prominent skin involvement in various autoimmune diseases, it would be of great interest to examine the effects of sex hormones on cell-type–specific gene expression in different autoimmune diseases that show sex bias. Skin involvement of lupus, termed CLE, is present in the majority of patients with SLE but can present also as a disorder on its own. There are multiple different types of CLE, and the exact pathogenesis for CLE is unknown, but increased type I IFNs are believed to be central to the development of CLE lesions (summarized by Turnier and Kahlenberg [2020]). Interestingly, sex bias in CLE is lower than in SLE, with reported female-to-male ratios of 4.5:1 for postpubertal disease onset (Dickey et al, 2013). Although it is known that estradiol contributes to the sex bias in SLE and likely in CLE, the mechanisms are not well-understood and require much additional research.

To summarize, besides their systemic effects on immune cells, estrogen and testosterone also have local effects on the skin (Table 1). Both the ERs and the AR are expressed in various skin cell types, including in keratinocytes and fibroblasts, and play important roles in many aspects of skin function, such as proliferation and wound healing. In particular, the effects on keratinocytes are of great interest because these have been shown to play a role in various sex-biased autoimmune diseases with a strong skin involvement, for example, SLE.

## A Role For Skin and Immunometabolism in Sex-Biased Autoimmune Diseases

As stated earlier, several systemic autoimmune diseases that show sex bias, such as SLE and SSc, are thought to have their origins in the skin. In SLE, patients frequently develop CLE prior to the development of systemic disease, and almost 90% of patients with SLE show involvement of skin at some point during the disease course (Fava and Petri, 2019). However, more research is required to understand how sex bias affects the skin, particularly during the early events in the development of autoimmunity involving aberrant stromal cell interactions and initial immune cell recruitment that ultimately evolve into systemic disease.

### The epidermis as a driver of systemic immune-mediated inflammatory disease

Currently, keratinocytes, the major constituent of the epidermal skin layer, have been implicated in development of different autoimmune diseases. Data from transgenic mouse models of lupus (Billi et al, 2019) and psoriasis (Billi et al, 2020; Gangwar et al, 2022) driven by gene overexpression in keratinocytes reveal that autoimmune and inflammatory skin disease can trigger systemic disease manifestations, including development of autoantibodies, immune complex deposition, and inflammatory joint disease. There is some evidence of similar mechanisms at play in humans—for example, sunburn can trigger SLE disease flares and has even been reported to precipitate life-threatening lupus nephritis (Schmidt et al, 2007). Furthermore, epidermal keratinocytes represent a major source of type I IFN in lupus, and this IFN-rich environment is capable of reprogramming immune cells to take on a pathogenic phenotype (Billi et al, 2022). Given the profound impact of sex hormones on gene expression in the skin and especially keratinocytes, it is quite plausible that sex hormones modulate systemic inflammatory disease in part through their effects on the skin environment.

### Sexual dimorphism in skin immune cells

Examining immunological differences in the skin of mice of both sexes, Chi et al (2024) recently identified an androgen–innate lymphoid cell type 2 (ILC2)–DC axis as the underlying cause for immune sexual dimorphism in the skin. They found a higher level of skin-resident T cells in female skin than in male skin. Adult male mice showed differences in immune skin cell composition, and castration of male mice prior to adulthood resulted in a skin immune cell composition similar to that seen in female skin. Skin DCs from female mice showed a more activated phenotype with enhanced migratory and T-cell–priming capabilities versus male skin DCs. These observations were dependent on male sex hormones because DC levels were increased by castration of males and decreased by testosterone injection of females. Female mice expressed a higher level of ARs and had a higher number of ILC2s in their skin than male mice, resulting in higher ILC2-derived cytokine levels than that in skin from males. Both castration and AR knockout abrogated these differences (Chi et al, 2024). These studies indicate an essential role for skin ILC2s in sex bias that could contribute to the differences in the skin immunocyte composition between females and males. The next step would be to verify whether this axis plays a role in human participants. If so, this would allow for therapeutic targeting in immune diseases with a strong skin component.

### VGLL3 as a sex-biased driver of autoimmunity in the skin

Besides sex hormones, other factors might also play a role in the development of skin manifestations in autoimmune diseases, either independently or in synergy with sex hormones. An earlier study by Liang et al (2017) identified VGLL3 as a transcription coregulator enriched in the skin of healthy women relative to that in healthy men. VGLL3 was found to drive a proinflammatory transcriptional program in skin that may predispose women to cutaneous autoimmune disease. Its expression was found to be higher in keratinocytes from normal skin of females and upregulated in various autoimmune diseases, including in keratinocytes from patients with SLE (Liang et al, 2017), fibroblasts from patients with SSc (Ma et al, 2024), and fibroblast-like synoviocytes in RA (Du et al, 2022). In the skin of healthy women, VGLL3 showed more prominent nuclear localization, consistent with higher transcriptional activity, whereas skin from healthy men showed indistinct localization. In skin biopsies from patients with lupus, VGLL3 localization was nuclear in both sexes, suggesting activation of this autoimmune pathway in disease regardless of sex. Substantiating a role for VGLL3 in sex-biased autoimmunity, overexpression of *Vgll3* in mouse epidermis—modeling an exaggerated version of human female skin—caused mice to develop skin lesions resembling discoid lupus and features of lupus-like systemic autoimmunity, including B-cell expansion, autoantibody production, immune complex deposition, and end-organ damage (Billi et al, 2019). Although many factors implicated in lupus pathogenesis were found to be upregulated by VGLL3, specific factors mediating VGLL3’s immunogenic effects have not been definitively identified nor have the factors regulating increased *VGLL3* expression in women or sex-specific subcellular localization of VGLL3.

Subsequent work has solidified the importance of VGLL3 in inflammation and autoimmunity, with identification of potentially pathogenic roles in fibroblast-like synoviocytes in RA as well as myofibroblasts and endothelial-to-mesenchymal–transitioning cells in SSc—2 autoimmune diseases that also exhibit striking female sex bias (Du et al, 2022; Ma et al, 2024; Pagenkopf and Liang, 2020; Takakura et al, 2021). This broader role in sex-biased autoimmune diseases suggests that VGLL3 could be an attractive therapeutic target; however, VGLL3 intersects with the Hippo pathway, a conserved signaling network that plays a central role in various cellular process required for cell proliferation and differentiation, organ growth, embryogenesis, and tissue regeneration/wound healing (Fu et al, 2022). VGLL3 competes with YAP1 for transcription factor–binding partners to drive gene expression (Hori et al, 2020). Studies are currently underway to identify the mechanisms and cofactors through which epidermal VGLL3 signals and contributes to the development of cutaneous and systemic autoimmune disease. It is worth noting that whereas VGLL3 seems to be enriched in the skin of women and is associated with female-biased autoimmune diseases, YAP1 is associated with pro-oncogenic signaling (Johnson and Halder, 2014; Morciano et al, 2021), and the incidence of cancer is higher in men (Kim et al, 2018). It would be of interest to investigate whether there is an association between YAP1 signaling in males that contributes to male predisposition to cancer. Although it is known that males develop more skin cancer than females (Adams et al, 2021) and that YAP1 is upregulated in many skin cancers (Howard et al, 2022), a direct connection between VGLL3 and YAP1 in skin cancer has not yet been identified. A better understanding of this balance between VGLL3 and YAP1 signaling would provide a potential for identification of drug targets. Our current understanding suggests the risk that VGLL3–TEAD inhibition may lead to unopposed pro-oncogenic signaling by YAP1–TEAD. Conversely, inhibitors of TEAD autopalmitoylation are currently entering clinical trials for certain advanced solid tumors (Barry et al, 2021). Several studies suggest that these are likely to inhibit YAP–TEAD but not VGLL3–TEAD function (Chan et al, 2016; Sun et al, 2022), risking unopposed proinflammatory signaling. TEAD inhibitors that function through other mechanisms—such as competitive inhibition using mimetic peptides based on the TEAD-binding domain of VGLL4 (Jiao et al, 2014)—may represent an approach for subverting the concern of disrupting the TEAD-dependent balance between autoimmunity and cancer. The Hippo pathway has other points of intersection with autoimmunity and inflammation (Kong et al, 2024), further complicating the prospect. It remains to be seen whether TEAD autopalmitoylation inhibitors present a true risk of inducing or unmasking latent autoimmune disease or whether this is only a theoretical threat.

In summary, recent research has identified an autoimmunity-prone transcriptional signature in the skin orchestrated by the female skin–enriched transcription coregulator VGLL3. In SLE, VGLL3 may be more active in both sexes. Transgenic mice with epidermal overexpression of VGLL3 develop lupus-like systemic disease and provide a tool for identifying therapeutic targets and investigating how epithelial–immune cell interactions precipitate systemic autoimmunity.

### Drivers of sexual dimorphism in skin immunometabolism

Beyond the broadly proinflammatory activity of VGLL3, another recent study implicated VGLL3 in nutrient sensing and thereby identified a potential role of VGLL3 in immunometabolism (Pagenkopf and Liang, 2020). Sex differences in immunometabolism have long been appreciated but have yet not been fully explored. Relevant to SLE, immunometabolism has been implicated in several aspects of this disease, including a role for mitochondrial alterations in T-cell metabolism. Inhibition of glutaminolysis, a key energy source for effector T cells such as T helper 17 (Th17) cells, has also been shown to impact glycolytic pathways and result in a reduction in Th17 cell differentiation in samples derived from patients with SLE (Kono et al, 2018). Furthermore, inhibition of glutaminolysis reduced the expression of hypoxia inducible factor-1α (HIF1α), a central player in Th17 development (Kono et al, 2019). These studies suggest that remodeling T-cell metabolism may enable restoration of normal T-cell development and thus could be a potential therapeutic approach for treating autoimmune diseases (Lin et al, 2024).

Lipid metabolism is fundamental for meeting cellular energy demands. Different immune cell subsets have different metabolic demands, with increased beta-oxidation of lipids for anti-inflammatory Tregs and increased glycolytic pathway activation for growth and proliferation of effector T cells (Gerriets and Rathmell, 2012). Dysregulated lipid metabolism has been strongly implicated in SLE at both the systemic and cellular levels, and both have been described in the context of cardiovascular comorbidities in SLE. It is of interest to assess whether and how sex affects these metabolic factors, especially because there is a significant difference between males and females with regard to lipid metabolism (Varlamov et al, 2014), which is at least partially controlled by sex hormones (Liang et al, 2022; Palmisano et al, 2018). In females, higher estrogen levels contribute to increased storage of white adipose tissue, whereas in males, testosterone contributes to higher muscle-to-fat ratios. However, white adipose tissue can act as an endocrine organ that plays a key role in regulating the immune system. Adipose tissue is involved in peripheral conversion of androgens to estrogens, and this may represent a means through which excess adiposity might predispose to autoimmunity. Multiple studies have demonstrated through Mendelian randomization that a higher level of body fat is a risk factor for development of various autoimmune diseases, including RA and psoriasis (Budu-Aggrey et al, 2019; Lindegård, 1986; Ogawa et al, 2019). Whether VGLL3 might influence the balance between lipogenesis and lipolysis in humans is unclear; however, research in mice and Atlantic salmon has identified a role for VGLL3 in regulating adipogenesis, further linking VGLL3 to energy balance (Ahi et al, 2024; Halperin et al, 2013).

Inflammatory skin diseases are metabolically hyperactive states that require increased nutrients. It was recently shown that the epidermis of inflammatory skin has increased expression of HIF1α and that this skews epithelial metabolism toward glycolysis (Subudhi et al, 2024). Mice lacking HIF1α in the epidermis exhibit reduced expression of genes involved in glycolysis, mitochondrial function, epidermal differentiation, and immunity (Subudhi et al, 2024). Furthermore, lactate production is reduced in the absence of HIF1α in the epithelium, and reduced lactate levels reduced the number of T cells, epidermal thickening, and IL-17A expression (Subudhi et al, 2024). Pharmacologic inhibition of either lactate receptors or lactate-metabolizing enzymes reduced the type 17 γδ T-cell response. This study illustrates the relevance of immunometabolism for cutaneous immunology and provides additional opportunities to study inflammatory autoimmune skin diseases. Future studies are needed to assess whether such metabolic mechanisms are affected by sex. Sex-specific targeted interruption of such immune–epithelial metabolic circuits will contribute to resolving sex-biased inflammatory epithelial diseases.

To summarize, discovery of roles for sex-biased factors such as VGLL3 in immunometabolism and uncovering the contributions of known immunometabolic factors such as HIF1α to epithelial immunity continue to deepen our understanding of the potential role of immunometabolism in sexually dimorphic immune responses in the skin.

## Looking Forward: Overcoming Challenges in Studying Sex Bias in the Future

Sex bias in autoimmune diseases represents the cumulative effect of genetics, transcriptional changes, hormonal effects, and environmental and social factors. Although it will be difficult to gain a comprehensive understanding of how these different factors interact to culminate in autoimmunity, this will be necessary for the development of more effective therapies, which are desperately needed. Male animal and human participants are overrepresented in preclinical and clinical trials owing to concerns that hormonal variability in female participants might obscure findings. This has resulted in a gap in our understanding of the effects of drugs on females. The practical implications include development of drugs that are more efficacious or more dangerous for one sex than for the other—differences that may not emerge until postmarketing surveillance. Many drugs have been identified that show greater efficacy in males. A well-studied example is TNF inhibitors, which show higher relative risk for remission in males with RA (Atzeni et al, 2009) and higher incidence of adverse effects in females with RA, resulting in higher rates of discontinuation (Souto et al, 2016). Similarly, men with ankylosing spondylitis showed greater improvement in disease activity scores than women after 12 weeks of anti-TNF therapy (van der Horst-Bruinsma et al, 2013). The United States Government Accountability Office reported that between 1997 and 2000, 8 of 10 drugs taken off the market had greater adverse effects in women (Klein and Flanagan, 2016). This clearly demonstrates the need to evaluate both sexes in clinical trials. Autoimmune disease clinical trials can present an unusual challenge in the difficulty of recruiting enough male patients for adequately powered analysis of this subgroup. This challenge must be met with special efforts for recruitment because men who have certain female-biased autoimmune diseases often present with more severe disease (Crosslin and Wiginton, 2011; Peoples et al, 2016; Runmarker and Andersen, 1993).

### Considerations for in vitro experiments

For in vitro experiments that utilize cell lines to test new drugs, the chromosomal sex of the cell lines is typically ignored. Furthermore, the ubiquitous use of plastic in laboratory experiments has been shown to skew results. Recent research found evidence that the chemical Bisphenol A—an endocrine-disrupting chemical with estrogen-like properties commonly found in plastic water bottles and animal cages—altered the effect of sex hormones on disease (Bruno et al, 2019). Similarly, the presence of indicator dyes can also act as endocrine disruptors. Although these variables are seldom considered in the laboratory, they may have unintended and unappreciated consequences in many experiments in a sex-specific manner and could affect early-stage drug development.

### Considerations for different stages in aging

Although appreciation for and understanding of the effects of immune aging have increased dramatically in the last decade, less is known about the immune changes that may occur during and after puberty and menopause. The coincidence of the average age of onset of several juvenile autoimmune diseases (Massias et al, 2020) with the average age of onset for puberty (Whincup et al, 2001) suggests that factors beyond aging contribute to alterations in the immune system and that the rise in sex hormone levels as seen in puberty is also involved. A recent systematic review of the bidirectional relationship between puberty and autoimmune diseases showed the poor documentation of these relationships and highlighted differences in disease outcome in those with onset before and after puberty (de Gruijter et al, 2021). In addition, disease symptom differences have been noted between different age groups of patients with SLE (Marder et al, 2015), with juvenile SLE having greater severity (Ambrose et al, 2016; Tucker et al, 2008). An explanation of the difference in disease severity between SLE age groups is still being sought. Intriguingly, VGLL3 has been linked to age at menarche and pubertal growth in male and female adolescents (Tu et al, 2015)—a role that appears to be ancient given similar findings in Atlantic salmon (Ayllon et al, 2015; Kjærner-Semb et al, 2018).

### Considerations for including female participants in omic-based studies and (pre)clinical trails

Over the last few decades, various omics studies have been performed to unravel biological processes and diseases, including autoimmune diseases. Although these studies significantly advanced our knowledge in this area, many of the omics-based studies provide no information on the sex of the subjects (Bond et al, 2021). Although posthoc sex identification may be possible on the basis of sex chromosome–specific transcript or protein detection, retrospective stratification by sex may still prove ineffectual owing to the failure to account for sex in the study design and lack of clear annotation. Stratifying such cohorts will lead to underpowered analysis and potential spurious findings or type II errors related to differences between the sexes. In the United States, the National Institutes of Health now requires the incorporation of sex as a biological variable in the design of all funded studies (Clayton and Collins, 2014), and the Horizon Europe program from the European Union intends to do the same (Accounting for sex and gender makes for better science, 2020). Implementing these approaches in future studies to assess the role of sex in autoimmunity will be important to identify the role of genes, proteins, signaling pathways, and various cell types contributing to sex bias in pathogenesis, manifestations, outcomes, and therapeutic responses. Future cohort studies should be designed in a sex-aware manner for sufficient statistical power for both sexes. The variable of gender and use of exogenous hormones is also important, and study participants should be offered the opportunity to contribute this information as well if they are comfortable doing so. As noted previously, females have been largely excluded from both animal and human clinical trials because of the hormonal fluctuations during the menstrual cycle, which make data more difficult to interpret and more variable, making these trails subsequently more expensive. However, a recent study has shown that inclusion of females in rodent studies does not increase variability in rodent research studies (Beery, 2018), which creates a premise for including more females in animal studies. This will improve preclinical assessments and data and will pave a more balanced path toward drug development for sex-specific healthcare.

Sex chromosome gene expression dynamics have been understudied for many reasons (summarized by Khramtsova et al [2019]). To name a few, RNA sequencing and microarrays have different sensitivity to transcripts with low expression, which affects the characterization of X chromosomal transcripts. Filters based on all genes disproportionally exclude X chromosomal transcripts, which can skew downstream analysis, results, and conclusions. The homology between the Y and X chromosomes may also affect mapping and variant calling. Some of these challenges may be addressed using new and improved tools such as the XYalign tool (Webster et al, 2019); however, because this is a fairly new tool, older RNA-sequencing data have most likely not been analyzed with this tool. In addition, owing to XCI, genotyping of the sex chromosomes has much lower accuracy than that of autosomes. This causes challenges for genome assembly and analysis as well as a lower intensity of an X chromosomal signal in males for array-based genotyping (Ritchie et al, 2011; Webster et al, 2019). Furthermore, there is a higher number of anomalies in the X chromosome than in autosomes and an underrepresentation of X chromosomal SNVs on standard arrays (Wise et al, 2013). Standard sequencing technologies do not determine which genetic variants are on the silenced X chromosome, making interpretation more complicated (Accounting for sex in the genome, 2017).

### Autoimmune disease and the pregnancy compensation hypothesis

The increase in the incidence of autoimmune diseases in recent decades is highest in places where increased use of contraception has resulted in reduced number of pregnancies. To address this association, one hypothesis that is being pursued is the so-called pregnancy compensation hypothesis. This hypothesis proposes that the female immune system has evolved to tolerate an immunologically invasive placenta while still defending against the assault of parasites and pathogens. This is thought to be enacted proximately through sex hormones and genetically through sex chromosome gene dosage. Changes in reproductive behavior—namely, decreased parity—in industrialized environments exacerbate the evolved sex differences, resulting in an increased risk of autoimmune disease observed in females, and a reduction in diseases such as cancer, which can be opposed by heightened immune surveillance. In other words, earlier and more frequent pregnancies might confer protection. This hypothesis also provides context for the benefits of pregnancy-associated hormones such as the placentally derived hormone estriol in autoimmune diseases (Palaszynski et al, 2004; Papenfuss et al, 2011; Sicotte et al, 2002). Although this hypothesis still requires further support, this evolutionary perspective offers a useful framework for understanding sex bias in autoimmunity.

## Conclusion

Much progress has been made in understanding how sex hormones and sex chromosomes contribute to sex bias in autoimmune disease development as well as the potential major role that skin has in these processes. Using improved and/or novel technologies, combined with inventive hypothesis-driven research, our understanding of the role of sex bias in autoimmune disease will contribute to improved and sex-specific drug development, reduced adverse events, and better treatments for autoimmune disease patients.

## ORCIDs

Vincent van Drongelen: http://orcid.org/0000-0002-2090-7617

Joanna Rew: http://orcid.org/0000-0003-4722-2017

Allison C. Billi: http://orcid.org/0000-0001-7115-9113

## Conflict of Interest

The authors state no conflict of interest.
